# The relationship between H19 and parameters of ovarian reserve

**DOI:** 10.1186/s12958-020-00578-z

**Published:** 2020-05-13

**Authors:** Xi Xia, Martina S. Burn, Yong Chen, Cengiz Karakaya, Amanda Kallen

**Affiliations:** 1 Reproductive Center, Peking University Shenzhen Hospital, Shenzhen 518000, China; 2 Division of Reproductive Endocrinology and Infertility, Department of Obstetrics, Yale School of Medicine, Gynecology, and Reproductive Sciences, New Haven, Connecticut 06512 USA; 3 Department of Histology and Embryology, School of Basic Medical Sciences, Fujian Medical University, Fuzhou, Fujian Province 350122 P.R. China; 4 Department of Medical Biochemistry, Gazi University School of Medicine, Ankara, Turkey

**Keywords:** H19, Noncoding RNA, ncRNA, Ovarian reserve, AMH

## Abstract

**Context:**

The H19 long noncoding RNA (lncRNA) belongs to a highly conserved, imprinted gene cluster involved in embryonic development and growth control. We previously described a novel mechanism whereby the Anti-mullerian hormone (*Amh)* appears to be regulated by *H19.* However, the relationship between circulating *H19* and markers of ovarian reserve including AMH not been investigated.

**Objective:**

To determine whether *H19* expression is altered in women with decreased ovarian reserve.

**Design:**

Experimental study.

**Setting:**

Yale School of Medicine (New Haven, USA) and Gazi University School of Medicine (Ankara, Turkey).

**Patients or other participants:**

A total of 141 women undergoing infertility evaluation and treatment.

**Intervention:**

Collection of discarded blood samples and cumulus cells at the time of baseline infertility evaluation and transvaginal oocyte retrieval, respectively.

**Main outcome measure:**

Serum and cumulus cell *H19* expression.

**Results:**

Women with diminished ovarian reserve (as determined by AMH) had significantly lower serum H19 expression levels as compared to controls (*p* < 0.01). Serum H19 was moderately positively correlated with serum AMH. H19 expression was increased 3.7-fold in cumulus cells of IVF patients who demonstrated a high response to gonadotropins, compared to low responders (*p* < 0.05).

**Conclusion:**

In this study, we show that downregulation of *H19* in serum and cumulus cells is closely associated with decreased ovarian reserve, as measured by decreased AMH levels and reduced oocyte yield at oocyte retrieval. Further study with expanded sample sizes is necessary to determine whether *H19* may be of use as a novel biomarker for diminished ovarian reserve.

## Precis

Downregulation of *H19* in serum and cumulus cells is associated with decreased ovarian reserve, as measured by decreased AMH levels and reduced oocyte yield at oocyte retrieval.

## Introduction

The problem of ovarian aging has considerable impact on public health, and presently no viable preventative or treatment options are available. With age, a drastic decline in the quantity of follicles and oocytes (the “ovarian reserve”), occurs [1], leading to decreased female fecundity and infertility. Historical data, as well as studies of women undergoing fertility treatment, have shown that female fertility begins to decline as early as age 30 [2, 3]. Women are increasingly delaying childbearing [4]. In 2014, over 30% of first births occurred in women over 30 [4]. Thus, more and more women are attempting to conceive in their later reproductive years, when fertility is already in decline [2]. Indeed, birth rates in the U.S. are at a record low [5]. Adding to the complexity of this challenge is the fact that fecundity varies even among women of similar age groups [6], and some women experience idiopathic accelerated follicle loss and early menopause. Why follicle growth and development go so drastically awry in some women is poorly understood, and many gaps remain in our understanding of the processes regulating the recruitment and growth of ovarian follicles.

To understand the factors underpinning these developments, it is essential to define the processes regulating the normal physiology of ovarian aging. In reproductive age women, the pool of primordial follicles is continuously depleted through cyclic recruitment. This relentless loss of follicles leads to a condition known as diminished ovarian reserve (DOR). Women with DOR are at increased risk for infertility and poor ovarian response to ovarian stimulation during in vitro fertilization (IVF) cycles [7].

We previously described a novel mechanism whereby follicular recruitment appears to be regulated in part by the long noncoding RNA *H19. H19* is highly conserved [8], expressed during embryogenesis and repressed in most adult tissues, with the exception of ovary, uterus, skeletal muscle and heart [9–13]. We showed that the ovaries of female *H19* knockout mice exhibit accelerated follicular recruitment and atresia and a more rapid decline in fertility as compared to WT mice [14] We also observed that *H19*KO mice produce less anti-Mullerian hormone (AMH) as compared to WT mice, a phenotype that is recapitulated in vitro in granulosa cells after *H19* knockdown [14]. AMH regulates the size of the follicular pool by inhibiting the initial recruitment of primordial follicles into the growing pool and modulating the sensitivity of growing follicles to follicle stimulating hormone, and is critical in the regulation of follicle numbers [15–17]. Mice lacking AMH exhibit early follicular recruitment and loss [16–18], and *AMH* sequence variants in humans have been associated with premature ovarian insufficiency (POI) [19].

AMH is an informative marker for the assessment of ovarian reserve, but its regulation remains poorly understood. Additionally, AMH is only one of several available markers of oocyte quantity. Poor ovarian reserve can be defined by multiple factors, including age, poor oocyte yield after IVF, and abnormal ovarian reserve testing markers including FSH^7^. A potential link between *H19* and markers of ovarian reserve in women, including AMH, has not yet been investigated. Given the relationship between *H19* and follicular recruitment we observed in mice, we sought to determine whether decreased circulating and follicular *H19* expression is linked to poor ovarian reserve, as determined by clinical and biochemical markers, (including but not limited to AMH), in a population of women with infertility.

## Materials and methods

### Collection of patient samples

In order to evaluate circulating *H19* expression and correlation with ovarian reserve in infertile women, discarded blood samples (2 mL) were collected from 69 patients undergoing evaluation for infertility at the Yale Fertility Center (New Haven, CT) (Table 1). Samples were collected in the early follicular phase (day 2–4 of the menstrual cycle). Serum was separated by centrifugation for 10 m at 12000 rpm at 4 °C and stored at − 80 °C until RNA extraction [20–22]. Patients were excluded if any of the following diagnoses were present: Cushing’s syndrome, hyperprolactinemia, adrenal hyperplasia, acromegaly, hypothalamic amenorrhea, hypothyroidism, or diabetes mellitus. Patients were divided into three groups: controls (women diagnosed with male and tubal factor infertility, *n* = 19), women with unexplained infertility (*n* = 24), and women with diminished ovarian reserve (DOR, defined as AMH < 1.1 ng/mL and antral follicle count < 5, *n* = 26). This study was approved by the Yale University Institutional Review Board (IRB protocol # 1606017946).

**Table 1:**
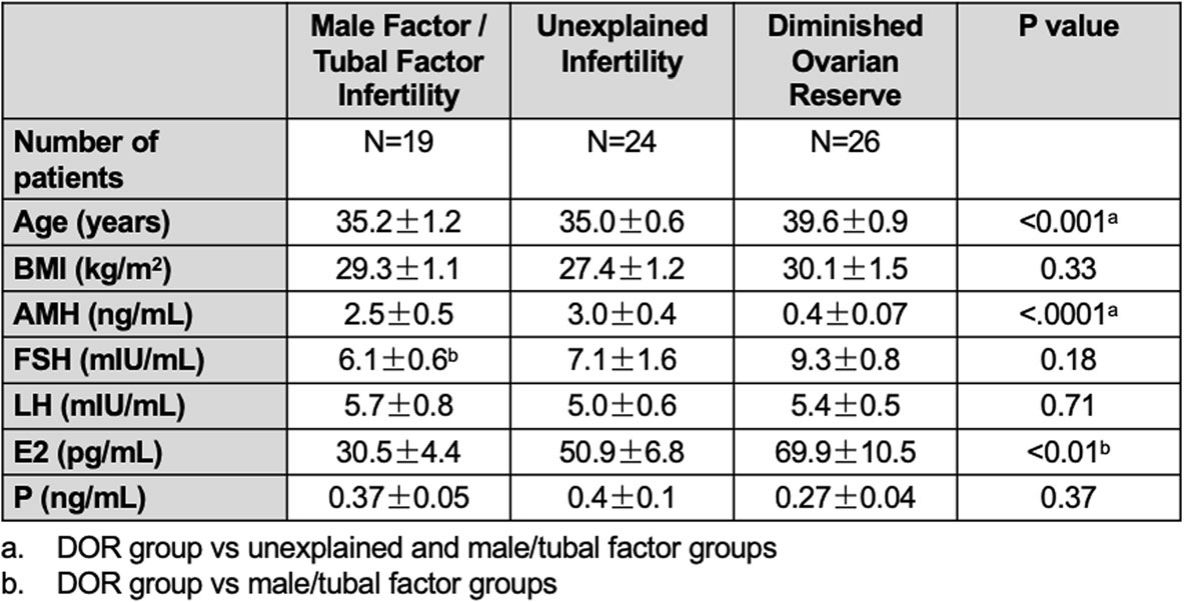
Descriptive statistics for patients from which serum samples were collected. Results of comparisons between baseline characteristics among the three groups are given. **a**.DOR group vs unexplained and male/tubal factor groups. **b** DOR group vs male/tubal factor groups

We also sought to determine whether *H19* is detectable in cumulus cells of women undergoing IVF with oocyte retrieval, and whether expression correlates with ovarian reserve as measured by response to gonadotropins. For these studies, we utilized pooled cumulus cells collected from a total of 72 consecutive cycles in women undergoing infertility treatment with IVF-ICSI at Gazi University School of Medicine IVF Center (Ankara, Turkey) (Table 2). Causes of infertility included diminished ovarian reserve, male factor, tubal factor, anovulation, endometriosis, and unexplained infertility, or a combination of these factors. For patients undergoing agonist cycles, treatment was initiated by GnRH agonists during the luteal phase of the preceding cycle. Stimulation with gonadotropins was initiated after downregulation had been achieved (estradiol level < 50 pg/ml in the absence of ovarian cysts on transvaginal sonography). For patients undergoing antagonist cycles, treatment with gonadotropins was initiated on cycle day 3 when serum progesterone was < 1 ng/ml and transvaginal sonography confirmed absence of ovarian cysts. A GnRH antagonist was added for pituitary suppression after five days of gonadotropin stimulation. Stimulation protocols included 150–300 IU/day of gonadotropins, either recombinant (GonalF, Merck) or in combination with human menopausal gonadotropin (hMG, Menopur, Ferring). Patients received hCG (Ovidrel 250 μg, Merck) when two or more follicles > 18 mm in diameter were present with an adequate estradiol response. Oocytes were collected 36 h after hCG injection. Retrieved cumulus-oocyte complexes (COCs) were placed in culture medium (G-MOPS, VitroLife) and cumulus cells were dissected from the oocyte mechanically in the absence of hyaluronidase. Cumulus cells from each patient were pooled in a single eppendorf tube. Samples were washed twice with 0.5 ml 1X Phosphate Buffered Saline (PBS) and centrifuged at 1000 x g for 1 min with the supernatant removed after each wash. After the final wash, the cell pellet was resuspended in 50 μl SideStep™ Lysis and Stabilization Buffer (Stratagene, La Jolla, CA). Samples were stored at − 80 °C until analysis.

**Table 2:**
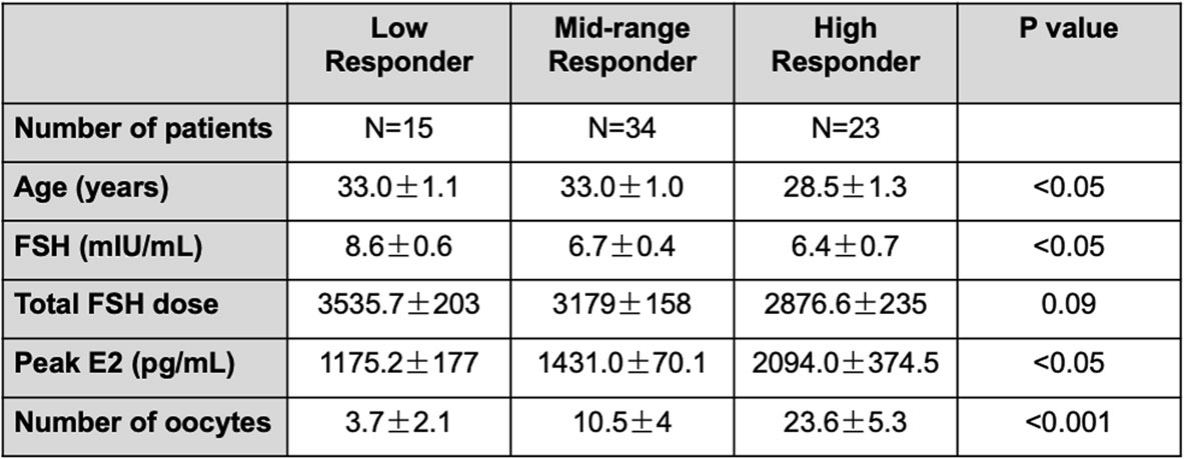
Descriptive statistics for patients from which cumulus cells were collected. Results of comparisons between baseline characteristics among the three groups are given

For study analysis, patients were stratified by percentiles based on the number of retrieved oocytes. The number of oocytes included in the first quartile, which correspond to percentile values lower than 25%, was considered as indication of poor response (PR) to controlled ovarian stimulation. Women producing more oocytes than the 75th % of their age group were considered high responders (HR) to controlled ovarian stimulation. Women whose oocyte production fell between 25-75th % of their age group were considered normal responders (NR). This study was approved by the Gazi University Institutional Review Board committee (IRB protocol #131/11.05.2011).

### Materials

Primers for H19 and β-actin were purchased from Real Time Primers (Elkins Park, PA, USA). The Norgen Plasma/Serum RNA Extraction Kit was purchased from Norgen Biotek (Ontario, CA). The Qiagen miRNeasy Mini Kit and RNeasy MinElute Cleanup Kits were purchased from Qiagen (Germantown, MD, USA). SYBRGreen was purchased from BioRad Laboratories (Hercules, CA, USA).

### RNA extraction, cDNA synthesis and RT-PCR

RNA extraction from stored blood samples was performed using the Norgen Plasma/Serum RNA Purification Mini Kit (Thorold, Ontario, Canada). To purify RNA from cumulus cells, the Qiagen miRNeasy Mini Kit and RNeasy MinElute Cleanup Kits were used according to the manufacturer’s instructions. RNA quantity and purity were determined using an ultraviolet spectrophotometer. Reverse transcription was carried out using the BioRad iScript cDNA synthesis kit (Hercules, CA, USA) in a 20ul reaction containing 0.5μg of total RNA. For cDNA synthesis, the reaction mixtures were incubated at 16 °C for 30 min, at 42 °C for 30 min, and at then 85 °C for 5 min. The cDNA was stored at − 80 °C until qPCR. qPCR was performed in a 25ul reaction containing 1.5ul of cDNA using Bio-Rad QSYBRGreen in a Bio-Rad iCycler. PCR was performed by initial denaturation at 95 °C for 5 min followed by 40 cycles of 30 s at 95 °C, 30 s at 60 °C, and 30 s at 72 °C. All SYBR green runs had dissociation curves to predict potential primer-dimers. Specificity was verified by melting curve analysis and agarose gel electrophoresis. The threshold cycle (Ct) values of each sample were used in the post-PCR data analysis, and the delta-delta Ct method was used to calculate mRNA expression. H19 levels were normalized and expressed as fold change relative to that of β-actin. The mean level of H19 expression in the control (tubal/male) factor group was set to 1, and H19 expression in the other two groups was normalized to this control group. All SYBR green runs had dissociation curves to predict potential primer-dimers. Specificity was verified by melting curve analysis. The PCR primers for the indicated genes are listed below.

Human H19 forward: 5′-GCACCTTGGACATCTGGAGT.

Human H19 reverse: 5′-TTCTTTCCAGCCCTAGCTCA.

β-actin forward: AAGAGCTATGAGCTGCCTGA.

β-actin reverse: TACGGATGTCAACGTCACAC.

### Statistical analysis

Descriptive statistics for all patients were analyzed with one-way ANOVA combined with Bonferroni post hoc analysis. To compare *H19* expression levels, one-way ANOVA was performed with Bonferroni post hoc analysis. Association between serum *H19* expression and AMH levels was measured using Pearson’s correlation coefficient analysis. All *P* values were considered to be statistically significant when *p* < 0.05. Statistical analysis was performed using GraphPad (Chicago, IL).

## Results

### H19 expression in serum

**Fig. 1:**
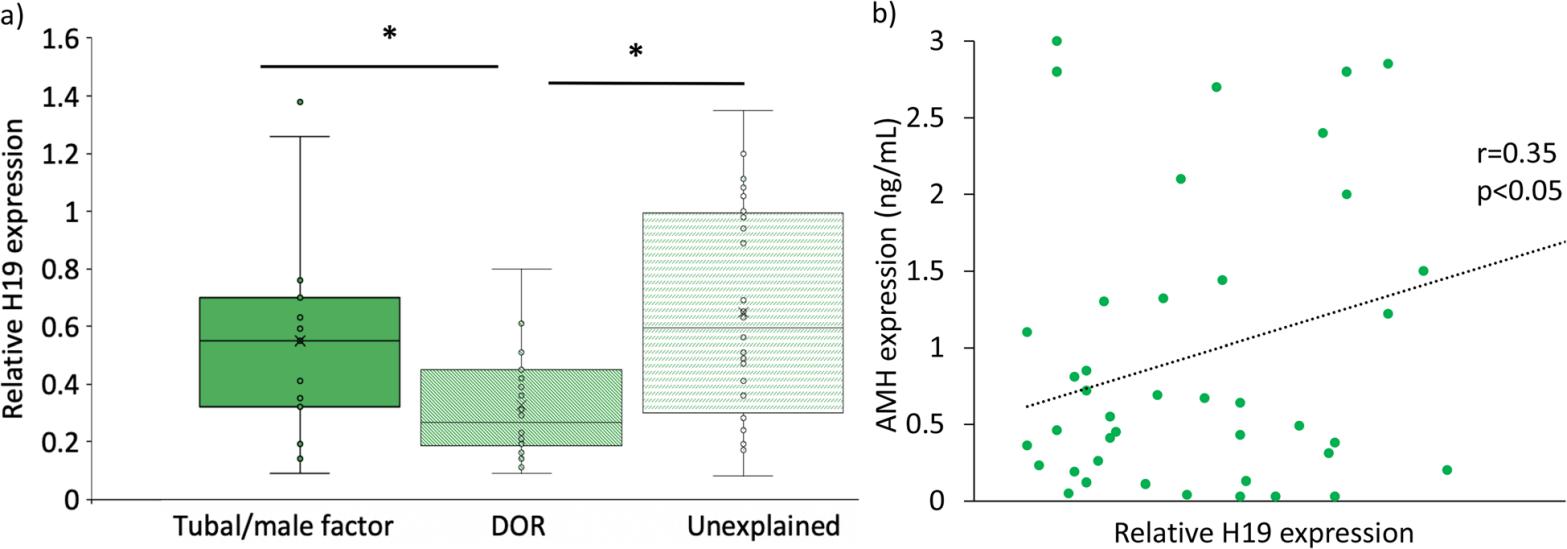
Serum *H19* expression in decreased in women with DOR. a) Expression level of *H19* in women with DOR is presented as fold change relative to women without DOR (i.e. unexplained infertility and male/tubal factor). Relative *H19* expression was decreased by half in women with DOR compared to women with male/tubal factor infertility, and by nearly 60% as compared to women with unexplained infertility (*p* < 0.05). B) A moderate positive correlation between *H19* expression and serum AMH levels (r = 0.35) was observed; p < 0.05

Women with DOR had significantly lower serum H19 expression level as compared to women with tubal factor infertility and women with unexplained infertility (Fig. 1a, *p* < 0.05). As expected, women with DOR also had significantly lower serum AMH levels 0.4 ng/mL for DOR vs 2.5 ng/mL for tubal factor and 3.0 ng/mL for unexplained infertility, *p* < 0.001). A moderate correlation was observed between serum *H19* expression and AMH (Fig. 1b, *p* < 0.05).

### H19 expression in cumulus cells

**Fig. 2:**
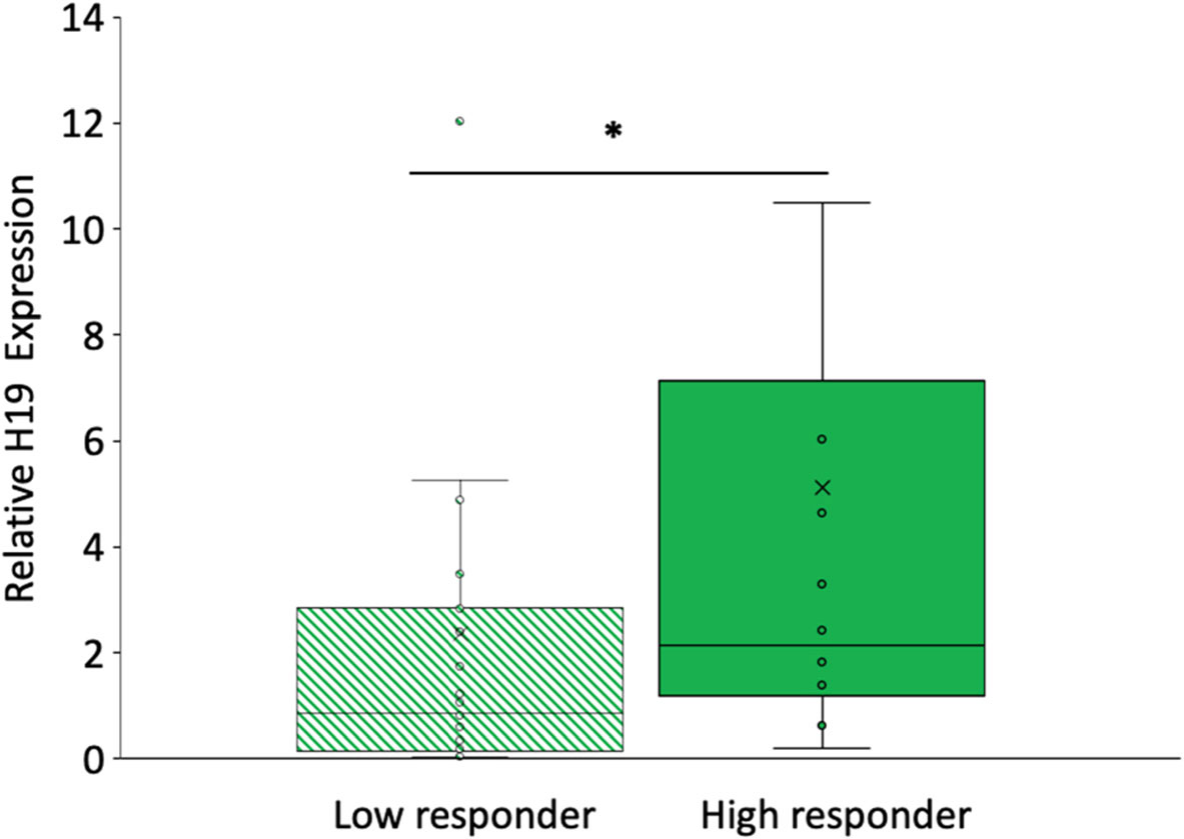
Serum *H19* expression in women who are poor responders to IVF treatment. Expression level of *H19* in women who were high and low responders to gonadotropins is presented as fold change relative to controls (women who had a normal / midrange gonadotropin response). Relative *H19* expression was significantly decreased in “low responder” women (p < 0.05)

H19 expression was significantly decreased (4.6 fold) in cumulus cells of IVF patients who demonstrated a poor response to gonadotropins compared to high responders (Fig. 2, *p* < 0.05). Women who were poor responders also had higher cycle day 3 FSH levels (8.6 mIU/mL vs 6.4 mIU/mL, p < 0.05) and lower peak E2 during IVF stimulation (1175.2 pg/mL vs 2094 pg/mL, *p* < 0.05). Total gonadotropin dose did not differ between the two groups.

## Discussion

In this study, we show that in women with evidence of decreased ovarian reserve, circulating and ovarian levels of *H19* are decreased. We observed that women with diminished ovarian reserve, as evidenced by a poor response to gonadotropins and a low oocyte yield after in vitro fertilization, also exhibited decreased cumulus cell *H19* expression. We also found that serum *H19* was lower in women with decreased serum AMH levels (a proxy for ovarian reserve which is predictive of low oocyte yield at retrieval [23]). We also observed a moderate, statistically significant correlation between decreased serum *H19* expression and decreased AMH. Altered expression profiles of microRNAs, another class of noncoding RNAs, has been linked with poor ovarian reserve in plasma and serum [24, 25], whole ovaries [26], follicular fluid [27], granulosa cells [28–30], and oocytes [31], and two studies have reported differential expression of piwi-interacting RNAs in GCs and oocytes from women with DOR [31, 32]. However, little is known about the role of long noncoding RNAs such as *H19* in ovarian aging.

More work is needed to determine whether *H19* may be useful as a marker of ovarian reserve alone or in combination with other markers, or as a prognostic marker for follicle loss remains to be determined. Currently available methods of determining ovarian reserve have their drawbacks. The antral follicle count, typically performed via transvaginal ultrasound, is invasive, uncomfortable, and subject to inter-observer variability. An early follicular phase FSH has poor sensitivity for predicting failure to conceive, and requires careful timing in order to be accurate. Estradiol has poor intra- and inter-cycle reliability and is useful only in conjunction with FSH [33]. Serum AMH has emerged as a widely used measure of ovarian reserve based on its consistency within and between menstrual cycles. AMH is useful as a predictor of ovarian response in controlled ovarian hyperstimulation cycles. However, the PPV in young women is poor, and AMH does not appear to predict time to pregnancy in non-infertile women [34]. Combining the different currently available ovarian reserve testing modalities does not appear to improve predictive ability over the use of a single test [35]. Importantly, all available tests are considered diagnostic rather than prognostic; we still seek the “holy grail” of a predictive test for follicle loss.

**Fig. 3:**
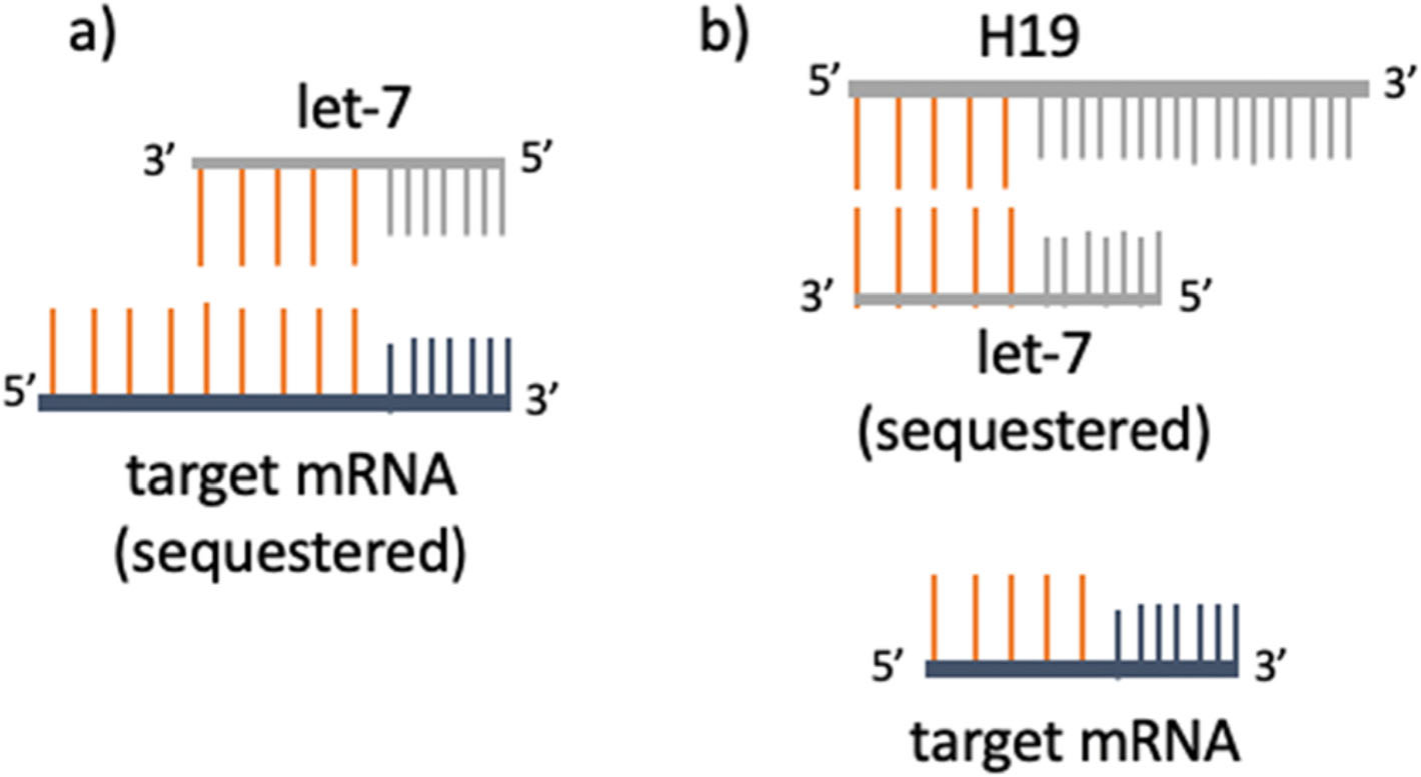
*H19* acts as a molecular “sponge” for let-7. (a) The microRNA let-7 functions as a negative regulator of target genes [36]. (b) We have shown that *H19* acts as a molecular “sponge” for let-7, binding and modulating its availability [36]. The *Amh* mRNA contains putative binding sites for let-7 [14], and let-7 transfection leads to decreased *Amh* expression in GCs [14], supporting *Amh* as a novel let-7 target and representing a novel ncRNA mediated mechanism by which the bioavailability of *Amh* can be regulated by *H19*

As a mechanistic explanation for how *H19* might regulate ovarian reserve, we have shown that in mice, loss of *H19* leads to accelerated follicular recruitment with increased spontaneous development of secondary, preantral, and antral follicles [14]. We also described a novel mechanism by which *H19* appears to regulate AMH. AMH is a critical “gatekeeper” which inhibits the activation of dormant ovarian follicles and slows the growth of maturing follicles [15–17]. Our previous work demonstrated low AMH, increased follicular recruitment and subfertility in *H19* knockout mice, suggesting a potential role for *H19* in the regulation of anti-Müllerian hormone AMH [14]. A possible mechanism for this can be found in a microRNA (miRNA) intermediary, let-7, which functions as a negative regulator of target genes [36]. We have shown that *H19* acts as a molecular “sponge” for let-7, binding and modulating its availability [36] (Fig. 3). The *Amh* mRNA contains putative binding sites for let-7 [14], and let-7 transfection leads to decreased *Amh* expression in GCs [14], supporting *Amh* as a novel let-7 target. Previous studies have demonstrated that let-7 expression is increased in GCs from women with poor ovarian reserve [30] and in plasma from women with premature ovarian insufficiency, a condition marked by early loss of ovarian follicles [25]. Taken together, these studies suggest a plausible ncRNA-mediated mechanism for AMH regulation by H19, via let-7, and points to a potential role for *H19* in ovarian aging by identifying a direct link between ncRNAs and follicular and oocyte quantity.

This work raises the crucial question of whether *H19* has a role in the regulation of ovarian aging. As expected, in order to examine whether *H19* is related to markers of ovarian reserve, our studies included a study population of women who were older than controls. One question is whether the decreased *H19* expression we have seen in these populations is correlated with aging itself. To our knowledge, no data exists regarding whether age-related changes in *H19* expression occur with time in reproductive tissues. Loss of *H19* has been linked to increased expression of markers of cellular aging and loss of cellular quiescence in hematopoietic stem cells and endothelial cells [37–39]. More work is necessary to clarify this question. However, even if the differences in *H19* expression observed between populations are a result of age, the idea that *H19* may represent a biological “rheostat” regulating the aging reproductive endocrine system beyond fertility is an intriguing one, especially given that ovarian aging has implications beyond fertility, including the serious health consequences of menopause such as vasomotor symptoms, osteoporosis, and cardiovascular disease.

The strengths of our study include the use of different testing modalities (serum and cumulus cells). Noncoding RNAs, including miRNAs and lncRNAs, can be collected from tissues as well as bodily fluids including plasma, serum, and urine [40, 41]. While tissue-specific changes in *H19* expression have been investigated as potential biomarkers in other conditions including breast [42] cancer, this is the first report of *H19* expression in cumulus cells, which are easily accessible at the time of oocyte retrieval. We also report a correlation between serum *H19* and AMH, a marker of follicular response during IVF^23^. Circulating *H19* has been explored as a diagnostic and prognostic biomarker for other conditions including coronary artery disease [43], multiple myeloma [44], and cancers including bladder and gastric cancer [22, 40, 41, 45, 46]. In the reproductive tract, circulating *H19* is higher in women with polycystic ovary syndrome (PCOS) compared to controls [47], and *H19* has been identified as a potential diagnostic and prognostic marker for epithelial ovarian [48] and cervical cancer [49]. However, a link between circulating *H19* and markers of ovarian reserve has not previously been described. Moreover, our sample groups include a diverse population of women at a range of ages and ethnic backgrounds, lending generalizability to our results. Our study is limited by the small sample size and the fact that limited demographic data and no AMH was available from the population of women from whom cumulus cells were obtained. However, while the finding of low *H19* in women with poor ovarian reserve may be correlative and requires further study, our findings regarding *Amh* and let-7 lend mechanistic plausibility to *H19* as a causative factor. Lastly, it is well established that imprinted genes, including *H19,* are susceptible to alteration by assisted reproductive technologies [50–52]. However, our findings of low *H19* in women with DOR are consistent across women undergoing controlled ovarian stimulation (COH) and those who presented prior to initiating COH. Additionally, our findings are consistent across different testing modalities (serum and cumulus cells).

In conclusion, our work suggests for the first time that circulating and intrafollicular H19 levels may be altered in women with diminished ovarian reserve. *H19* may have an important role as a master regulator of ovarian reserve markers, particularly AMH, and further elucidation of the role of *H19* in the ovarian aging process will enhance our understanding of normal folliculogenesis as well as the pathogenesis of diminished ovarian reserve. Further studies should be conducted to determine whether the association we have observed holds true in younger women with diminished ovarian reserve. In the future, *H19* may also prove useful as a novel diagnostic biomarker for ovarian reserve and premature ovarian insufficiency, and has the potential to improve our diagnosis of DOR especially in situations where current ovarian reserve testing has proven inadequate.

## Data Availability

Data supporting findings are presented within the manuscript.

## Abbreviations

AMH: Anti-mullerian hormone
COH: Controlled ovarian hyperstimulation
DOR: Diminished ovarian reserve
E2: Estradiol
FSH: Follicle stimulating hormone
GC: Granulosa cell
hCG: Human chorionic gonadotropin
hMG: Human menopausal gonadotropin
IVF-ICSI: In vitro fertilization – intracytoplasmic sperm injection
lncRNA: Long noncoding RNA
miRNA: microRNA
ncRNA: noncoding RNA
PPV: Positive predictive value
